# Total hip/knee arthroplasty in the treatment of tumor-induced osteomalacia patients: More than 1 year follow-up

**DOI:** 10.1371/journal.pone.0177835

**Published:** 2017-05-17

**Authors:** Wei Zhu, Qi Ma, Yanyan Bian, Qianyu Zhuang, Zenan Xia, Jin Jin, Xisheng Weng

**Affiliations:** Department of Orthopaedics, Peking Union Medical College Hospital, Chinese Academy of Medical Sciences & Peking Union Medical College, Beijing, China; Mayo Clinic Minnesota, UNITED STATES

## Abstract

**Background:**

Tumor-induced osteomalacia (TIO) may result in a better prognosis after complete resection of the causative neoplasm. However, tumors located proximal to the articular surface of the metaphysis remain largely uninvestigated.

**Methods:**

A retrospective study of sixteen patients was undertaken to evaluate treatment of tumors with joint arthroplasty and tumor resection. The bone metabolism index, hip/knee joint function, arthroplasty complications and symptoms were followed up for at least 12 months in each patient.

**Results:**

All patients presented with neoplasms situated in the articular surface of the metaphysis, with 13 cases undergoing hip arthroplasty and 3 undergoing knee arthroplasty. Treatment of the tumors with joint arthroplasty and tumor resection significantly and rapidly ameliorate bone metabolism indexes in patients with TIO (p<0.01), with no identified tumor recurrence. The joint function evaluation score was improved in 15 patients (93.75%). Complications in these patients included post-operative pain, joint squeaking and secondary hyperparathyroidism.

**Conclusions:**

Joint arthroplasty that includes tumor-expanding resection appears to be a safe and appropriate method for the treatment of TIO patients with a neoplasm located in the metaphysis proximal to the articular surface.

**Level of evidence:**

Therapeutic Level III. See Instructions for Authors for a complete description of levels of evidence.

## Introduction

Tumor-induced osteomalacia (TIO), a rare paraneoplastic syndrome resulting from phosphaturic mesenchymal tumor mixed with connective tissue, is characterized by renal phosphate leaking and deranged bone turnover [1]. TIO usually induces diffused bone pain, hypophosphatemia, osteoporosis, loss of height and other symptoms.

Surgical treatment to completely resect the neoplasm leads to an excellent prognosis [2]. However, unique tumor areas have added complexity to this surgery, such as neoplasms located in the femoral head or tibial plateau. Complete tumor resection near the joints usually leads to destruction of the articular surface. The difficult situation induced during the operation of these unique areas greatly affects the function of the joint, which undergoes resection and cement construction without arthroplasty.

Joint arthroplasty on tumor-induced osteomalacia may be substantially different than that on a normal patient due to the osteoporosis and bone pain resulting from renal phosphate leaking [3]. Tumor resection could also be involved in the process of osteotomy. No clinical studies have been performed to determine the safety and effectiveness of joint replacement in tumor-induced osteomalacia patients.

Whether joint arthroplasty is the appropriate method when treating special tumor sites has not been determined. Our study included 16 patients diagnosed with tumors located near the hip or knee joint to analyze this treatment method. All patients underwent joint arthroplasty after tumor resection and had more than 1 year of follow-up.

## Materials and methods

### Patients

This retrospective study included 16 patients who were diagnosed with tumor-induced osteomalacia in our hospital and then underwent joint arthroplasty from December 2004 to December 2015. All participants were recruited to this study in July 2016. This study was conducted from June to November, and data were collected from July to August in 2016. We had access to information that could identify individual participants during or after data collection. Our hospital’s ethics committee reviewed the design of this study and deemed that this study was exempt from the requirement for ethical approval. We also obtained informed consent from all patients.

The inclusion criteria were adult patients with a tumor-induced osteomalacia diagnosis, a tumor located in the metaphysis around a hip or knee joint with poor function, no secondary tumor area, tumor resection followed by joint prosthesis replacement, and at least 12 months of follow up. The exclusion criteria were patients who could not be diagnosed with tumor-induced osteomalacia, those who underwent non-surgical treatment or that without joint arthroplasty, those who had more than one tumor location and a follow-up of less than 12 months.

### Diagnosis

All suspected TIO patients underwent evaluation with serum phosphorus [P], serum calcium [Ca], serum 1,25-[OH]2D, serum alkaline phosphatase, and serum parathyroid hormone. ^99^Tc^m^-octreotide (OCT) scanning and ^68^Ga DOTA-TATE PET/CT were conducted for functional imaging. X-ray, CT, and magnetic resonance imaging (MRI) were used to confirm the anatomic location. Above is the stepwise diagnostic approach that we previously reported [4]. Patients with a tumor located on the metaphysis and poor joint function were recommended to undergo arthroplasty.

### Surgical procedures

For all patients, general anesthesia and intravenous antibiotics were administered 30 min prior to surgery. Each patient exhibited a phosphaturic mesenchymal tumor located near a hip or knee joint. An experienced surgeon exposed the tumor area by classifying the soft tissue and conducting tumor segmental resection according to the anatomic location of the tumor. Extended local excision around the tumor was conducted to adjust the prosthesis implantation. Due to the special area near the joint, for example the femoral head or tibial plateau, osteotomy was followed with an expanding area to match the lines of force. For joint function, hip or knee joint prostheses would be implanted. Cement may have then been used to fasten the prosthesis. The wound was sewn carefully, and a drainage tube was reserved.

### Post-operative monitoring and follow-up

After the operation, serum phosphorus, calcium and alkaline phosphatase were routinely detected within 7 days in all patients to evaluate the effects of surgery. The patients were asked to return to our orthopedic department for follow up for at least 12 months. Symptoms, X-ray and bone mineral density were regularly monitored throughout the follow-up period. To assess joint function, the Knee Society Score (KSS) and HARRIS score were evaluated after surgery 12 months.

### Statistical analysis

All data were analyzed using SPSS version 11.0 (SPSS Inc., Chicago, IL, USA). Quantitative data for age, tumor diameter, BMI index, and follow-up time are expressed as the means±SD. Serum phosphorus, serum calcium, alkaline phosphatase and joint function score before and after surgery were assessed using a paired T-test. P-values lower than 0.05 were considered significant.

## Results

### Demographic and clinical characteristics of TIO patients

**Fig 1:**
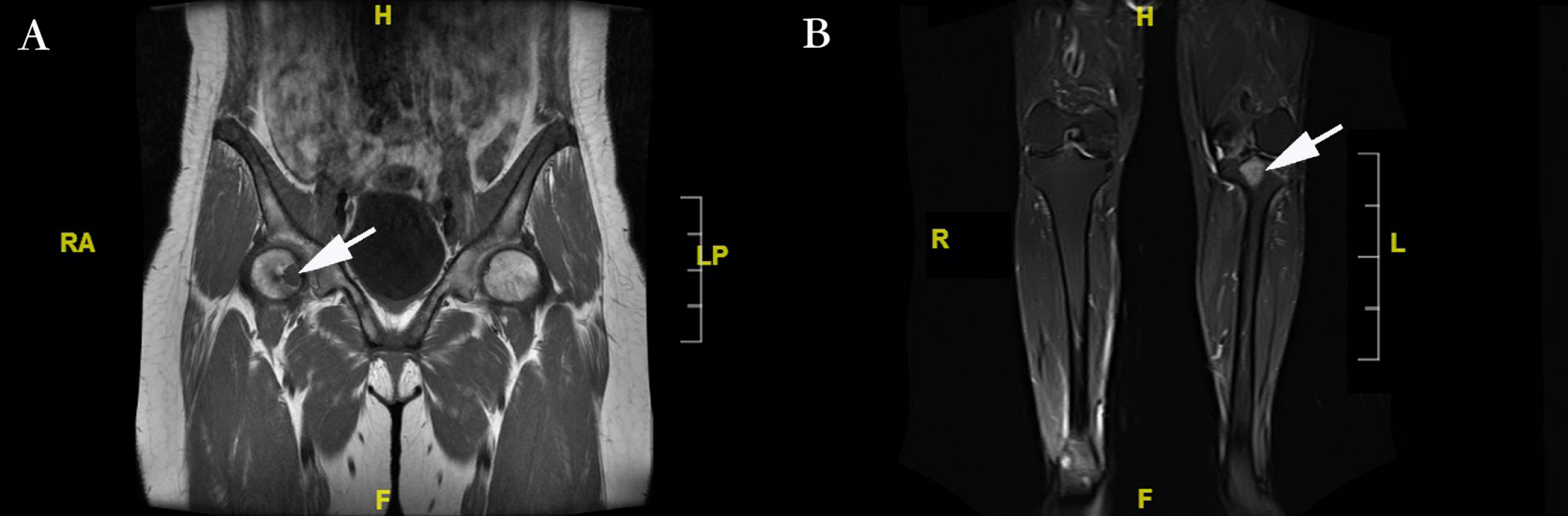
The causative lesion of TIO, as shown by MRI to identify the particular location. **1A.** A 24-year-old female patient with tumor-induced osteomalacia has a lesion located in the right femoral head. The arrow represents the tumor area. **1B**: Causative lesion located in the tibial plateau of a 51-year-old male tumor-induced osteomalacia patient. The white arrow represents the tumor area.

**Fig 2:**
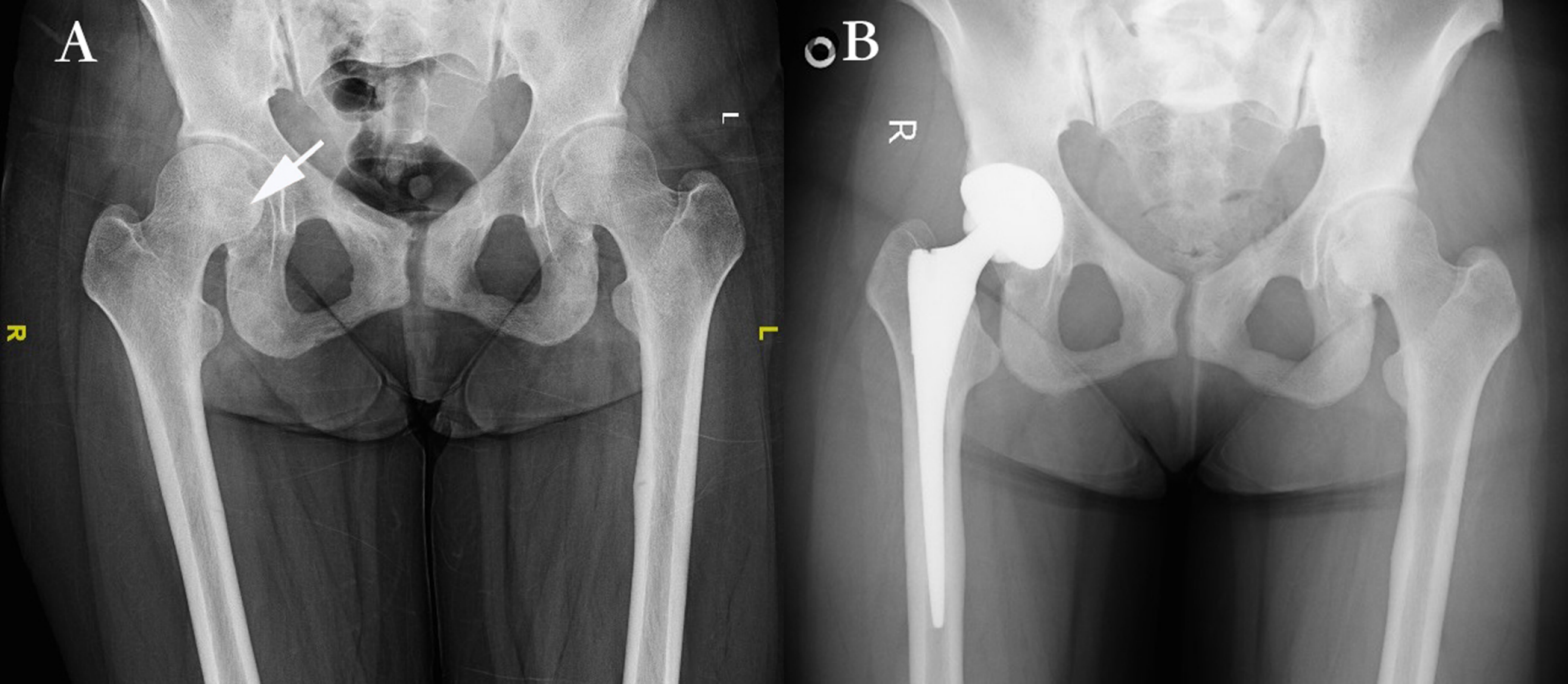
X-ray examination before and after joint arthroplasty in the treatment of tumor-induced osteomalacia patients. **2A**: Osteoporosis and the causative lesion in a 52-year-old male TIO patient are shown on X-ray before joint arthroplasty. **2B**: The patient underwent joint arthroplasty after one year. Osteoporosis was significantly improved, as shown by X-ray.

**Fig 3:**
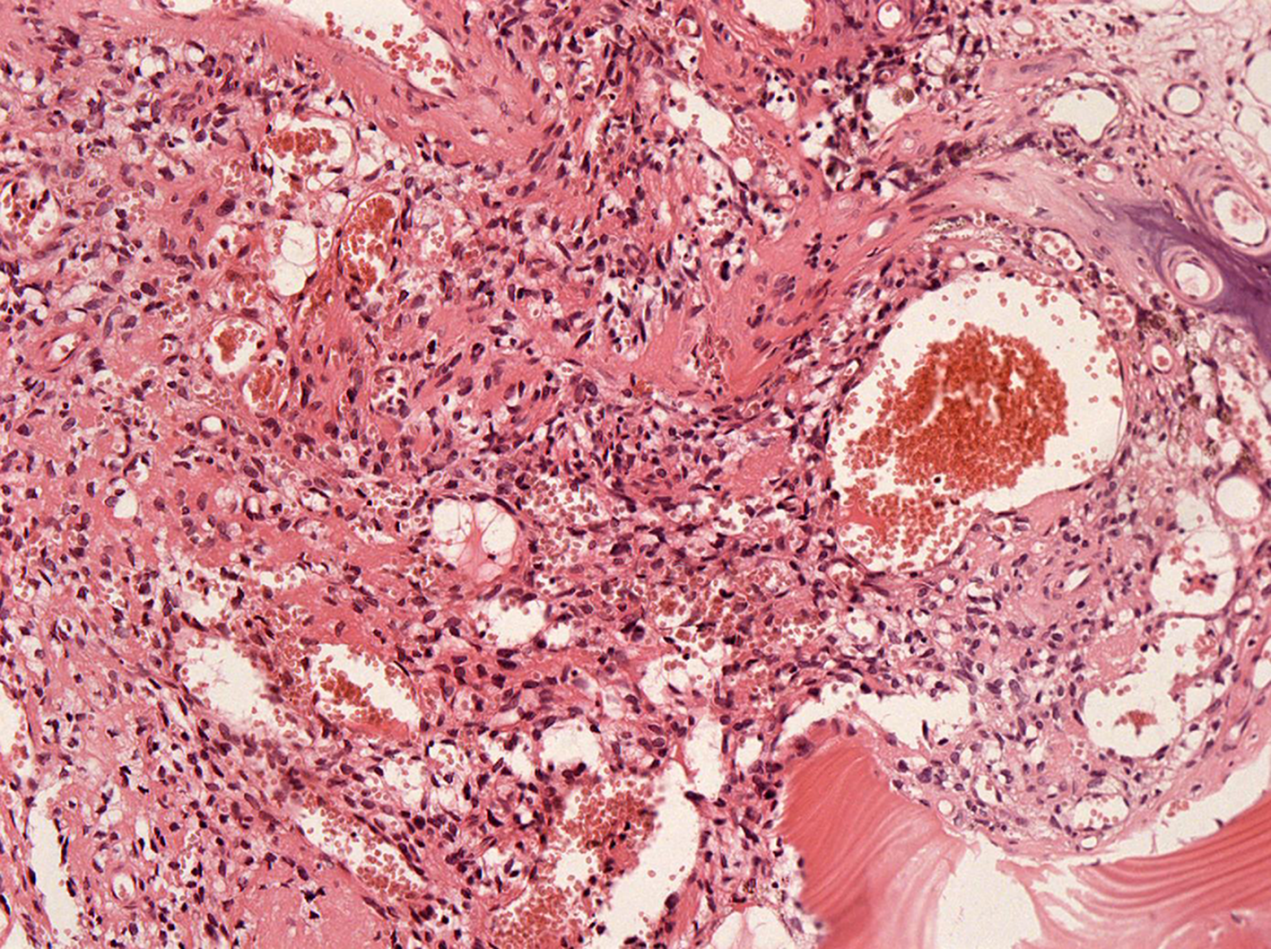
Pathological results of the patients. Histopathological examination revealed that the neoplastic cells were spindled to stellate in shape with elaborate intrinsic microvasculature, infiltrating between bone trabecular (HE, 100x).

As shown in Table 1, sixteen TIO patients with special tumor locations were involved in this study, which was composed of 10 males (62.5%) and 6 females (37.5%). The age of these patients ranged from 24 to 60 with an average of 44.67±9.58 years. The BMI in these patients ranged from 18.73 to 35.86. Tumor locations in these patients were atypical; 11 patients had tumors situated in the femoral head (**Fig 1**) and 2 in the tibial plateau. The tumor of one patient was located on the left femoral condyle and proximal fibula. One patient’s tumor was situated in the right femoral trochanteric. The mean maximum diameter of these uniquely located tumors was 2.04±1.56 cm. The follow-up time after surgery was 2.86±1.56 years. The period from diagnosing TIO to joint arthroplasty was highly heterogeneous in these patients, ranging from 0.5 months to 336 months with an average of 62.33 months. All patients received tumor resection followed by joint prosthesis arthroplasty in our study, as shown in **Fig 2**. The pathological results of the patients are presented in **Fig 3**.

**Table 1:** Characteristics of TIO patients with joint arthroplasty.

NO.	Male/Female	Age	BMI	Tumor area	Tumor Maximum Diameter (cm)	Follow-up time(years)	Diagnosis of TIO to operation(months)
1	M	39	24.2	Right femoral head	1.7	6.4	44.4
2	M	51	27.61	Left tibial plateau	2.1	4.3	0.67
3	M	48	24.34	Right femoral head	0.6	2.5	60
4	M	57	18.73	None	N/A	1.1	19.2
5	M	52	26.45	Right femoral head	3	1.2	84
6	M	46	24.4	Left femoral condyle and proximal fibula	1	1.5	0.5
7	M	46	35.86	Right femoral head	1	3.5	336
8	F	32	25.72	Right femoral head	N/A	2.1	24
9	F	35	22.21	Left femoral head	1	2.9	12
10	F	47	23.4	Right femoral head	3	4.6	0.81
11	M	37	25.61	Right femoral head	1.9	2.6	2
12	F	48	31.3	Left femoral head	2.5	2.25	1
13	F	24	26.44	Right femoral head	1.5	2.25	102
14	M	53	30.27	Right femoral trochanteric	6.8	1	186
15	M	40	21.1	Left femoral head	1	2	48
16	F	60	20.5	Left tibial plateau	1.5	5.6	84

### The influence of TIO on joint arthroplasty

The osteomalacia induced by the phosphaturic mesenchymal tumor raises the issue of whether joint arthroplasty would be as useful as that on other patients. Before surgery, the primary symptoms in our study included bone pain, fatigue and difficulty walking and osteoporosis, which all resulted from TIO (**Table 2**). Sixteen TIO patients involved in this study underwent total joint replacement after special area tumor resection. Thirteen patients underwent total hip arthroplasty (10 right and 3 left), and the other 3 patients underwent total knee arthroplasty on the left leg. The types of implanted prosthesis are presented in **Table 2**.

**Table 2:** The follow-up of arthroplasty with influence of TIO.

NO.	Pre-operative clinical Symptoms	Symptom follow-up after operation	Surgery	Prosthesis type	Pre-operation KSS/HARRIS score	Post-operation KSS/HARRIS score	Complications
1	Bone pain, fatigue	Relieved	THA(R)	Smith&Nephew: Reflection, Alumina, Synergy	52	98	None
2	Bone pain	Relieved	TKA(L)	Knee Prosthesis, Chunli Zhengda, China	63	85	Still bone pain
3	Difficulty standing, bone pain, fatigue, alopecia	Relieved	THA(R)	Smith&Nephew: Reflection, Alumina, Synergy	55	97	None
4	Bone pain, fracture	Relieved	THA(L)	DePuy: Pinnacle, Corail, BIOLOX	35	87	None
5	Difficulty standing, bone pain, dorsal paresthesia	Relieved	THA(R)	DePuy: Pinnacle, Corail, BIOLOX	64	93	Joint squeaking
6	Bone pain, difficulty walking	Relieved	TKA(L)	DePuy: Pinnacle, Corail, BIOLOX	50	78	Secondary hyperparathyroidism
7	Bone pain, fatigue, difficulty walking	Relieved	THA(R)	DePuy: Pinnacle, Corail, BIOLOX	66	90	None
8	Bone pain, bone shortening, difficulty walking	Relieved	THA(R)	ZIMMER: TMq+VerSys, Ceramics-Polyethylene	74	88	None
9	Bone pain, fatigue, difficulty walking	Relieved	THA(L)	Smith&Nephew: Reflection, Alumina, Synergy	62	92	None
10	Bone pain, fatigue	Relieved	THA(R)	Smith&Nephew: Reflection, Alumina, Synergy	45	90	Prosthesis long pain
11	Bone pain, fatigue, difficulty walking	Relieved	THA(R)	DePuy: Pinnacle, Corail, BIOLOX	70	98	Still bone pain
12	Bone pain	Relieved	THA(L)	ZIMMER: TMq+VerSys, Ceramics-Polyethylene	40	85	None
13	Bone pain	Relieved	THA(R)	Smith&Nephew: Reflection, Alumina, Synergy	59	88	None
14	Bone pain, difficulty walking	Relieved	THA(R)	Smith&Nephew: Reflection, Alumina, Synergy	30	69	None
15	Bone pain, fatigue	Relieved	THA(R)	ZIMMER: TMq+VerSys, Ceramics-Polyethylene	45	81	None
16	Bone pain, bone shortening	Not Relieved	TKA(L)	Knee Prosthesis, Chunli Zhengda, China	46	32	Still bone pain, walking restrict

#### Joint function assessment

**Fig 4:**
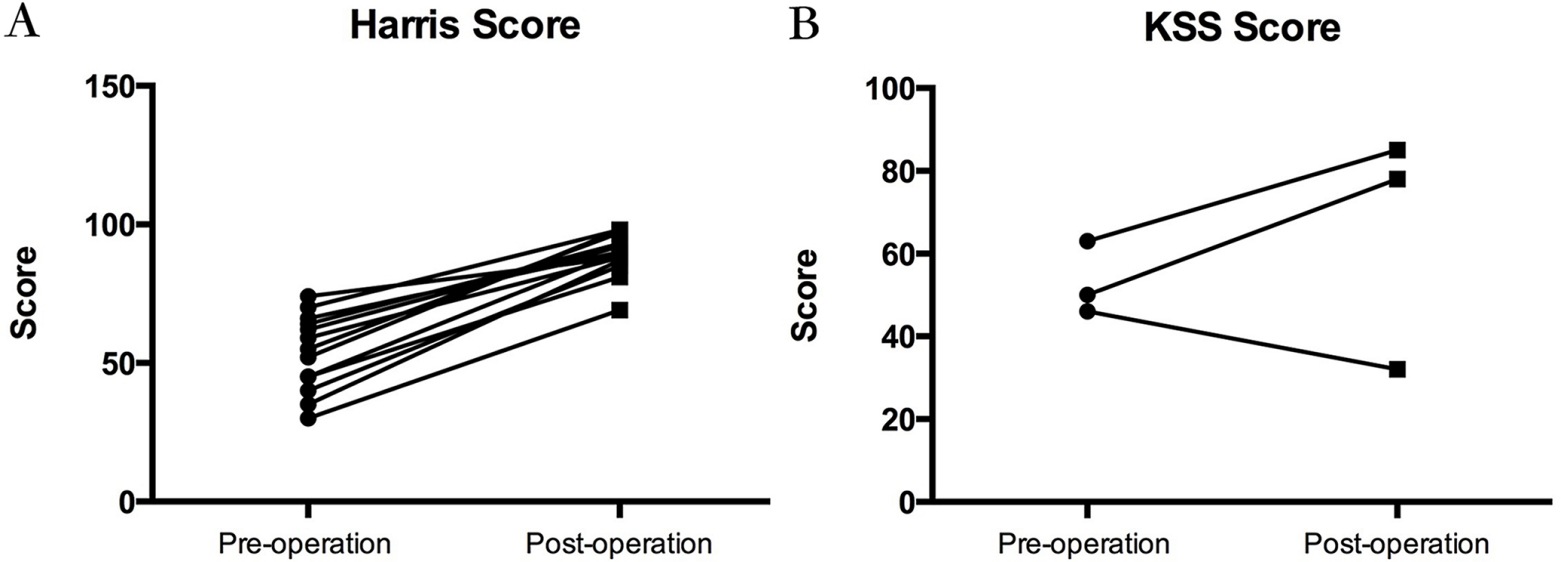
Harris/KSS score for assessing joint arthroplasty before and one-year after surgery. Thirteen patients underwent hip arthroplasty; changes in joint Harris score are shown in **4A**. Three patients underwent knee arthroplasty; changes in joint KSS score are shown in **4B**.

Twelve months after the surgery, joint function in the prosthesis-implanted side was assessed using the Harris/KSS score. As shown in **Table 2**, the post-operation score significantly improved compared with that before operation. Most patients were able to walk and had improved quality of life. In all 13 hip joint arthroplasty patients, the Harris score consistently increased (**Fig 4A**, p<0.01). However, with the exception of one patient with TKA, the KSS score decreased after surgery. Another two patients showed an increased function assessment score (**Fig 4B**). A tumor near the knee joint led to a slight difference.

#### Complications

Complications in this group of patients are shown in **Table 2**; 10 of 16 (62.5%) patients did not experience any complications within 12 months after surgery. Three patients (18.75%) continued to show bone pain after the operation. One patient had lengthy prosthesis pain (at least 3 months). Only one patient (6.25%) had a walking restriction. Another patient (6.25%) had joint squeaking after arthroplasty. Another patient exhibited secondary hyperparathyroidism after the operation and received another surgery for parathyroid removal. No patients exhibited complications such as hemorrhage, prosthesis infection, prosthesis loosening or TIO recurrence. During follow-up, the 3 patients who underwent knee arthroplasty had complications, while only 3 of 13 hip arthroplasty patients had complications.

### Serum phosphorus, calcium and alkaline phosphatase dynamic changes

To assess the effects of arthroplasty on TIO, the dynamic bone metabolism index of these patients was detected pre- and post-operatively. As shown in **Table 3**, alkaline phosphatase significantly decreased 7 days after the operation compared with that before the operation (p<0.01). Serum phosphorus showed an obvious increase after tumor resection (p<0.01). Most serum phosphorus levels quickly returned to normal within 1 week after surgery. However, serum calcium showed few decreases as the serum phosphorus dynamic increased (p<0.01). These bone metabolism indexes indicated that the body could rapidly recover after tumor resection and arthroplasty within several days.

**Table 3:** Dynamic changes in bone metabolism indexes.

NO.	Alkaline Phosphatase (U/L)		Serum Phosphorus (mmol/L)		Serum Calcium (mmol/L)		Osteoporosis Pre-operation	Osteoporosis at 1 year follow-up
	7 day Pre-operation	7 day Post-operation	7 day Pre-operation	7 day Post-operation	7 day Pre-operation	7 day Post-operation		
1	160	139	0.62	1.28	2.4	2.17	-3.3	Becoming normal
2	255	178	0.42	0.61	2.34	2.15	-4	Becoming normal
3	161	139	0.35	0.88	2.07	1.79	-2.7	Becoming normal
4	92	91	1.13	1.2	2.44	2.09	N/A	N/A
5	532	430	0.47	1.21	2.28	2.09	N/A	N/A
6	311	214	0.32	0.79	2.86	2.64	-3.4	Still present
7	77	81	0.65	0.84	2.13	2.1	-2.4	Becoming normal
8	323	164	0.48	1.1	2.38	1.94	-4.2	N/A
9	122	120	0.4	1.08	2.13	2.02	-2	N/A
10	184	151	0.27	1.58	2.19	2.04	-3.1	Becoming normal
11	413	339	0.44	0.82	2.21	1.98	-2.9	Becoming normal
12	133	128	0.44	0.83	2.25	2.18	-2	N/A
13	190	98	0.41	0.92	2.25	2.13	N/A	N/A
14	154	102	0.57	1	2.28	1.99	-6.9	Still present
15	259	244	0.53	1.04	2.21	2.08	-4.2	Still present
16	108	98	0.61	1.06	2.32	2.09	-2.4	Still present
P value		0.001		<0.000		<0.000		

### Osteoporosis and clinical symptom follow-up

Osteoporosis induced by TIO disease is characterized by continuous renal phosphate leaking and greatly influences quality of life. We evaluated the prognosis of osteoporosis and its resulting symptoms after tumor resection surgery. **Table 3** shows that most of our patients exhibited osteoporosis. During the 1-year follow-up after surgery, bone density normalized in 6 patients. Four patients continued to suffer from osteoporosis but have experienced symptom relief compared with before. The height of 1 patient (6.25%) increased because the waist had straightened. Before the operation, bone pain and difficulty walking were the primary symptoms. Fifteen of 16 patients (93.75%) had these symptoms relieved after tumor resection and arthroplasty, especially regarding bone pain. Most patients could walk and run with improved joint function (**Table 2**). These outcomes demonstrate a better effect of arthroplasty on TIO patients with unique tumor locations.

## Discussion

TIO, an extremely rare disease that leads to great suffering and is an important cause of hypophosphatemia [5,6], usually receives a good prognosis if the tumor is completely resected. Due to its rarity, few studies have focused on the surgical treatment strategy to achieve a better prognosis [4]. A tumor located on the long bone metaphysis, especially in unique areas such as the femoral head or tibial plateau, lack surgical guidelines. Studies have found that tumor arthroplasty has played a crucial role in functional rehabilitation, quality of life and daily activities, and treatment patterns depend on the size of the tumor and its localization [7,8].

Drawing from a large Chinese population, sixteen TIO patients with tumors situated around a joint were collected to evaluate joint arthroplasty. Arthroplasty treatment was very effective as a curative method under these special conditions and contributed to changes in joint function, complications and osteomalacia.

KSS/Harris score assessments and other follow-up measures showed that 15 of 16 patients (93.75%) experienced effective joint function improvement. One patient felt extreme pain around the left knee prosthesis and could not walk after surgery. This may be explained by residual tumor near the knee, which would lead to recurrence of TIO. Besides, we also found that this patient did not take anti-osteoporotic regularly and not participate in rehabilitation exercise actively after surgery. These behaviors were obviously not helpful for the recovery. Most patients had lost or relieved bone pain symptoms. Activity of daily living was greatly improved. Ten of 16 patients (62.5%) had no complications during the 12 months after surgery. Bone pain was the most important complication after surgery and was also one of the most common conditions occurring after osteoarthritis arthroplasty [9].

In this study, tumors near the hip joint were more common than those near the knee joint. However, hip joint arthroplasty showed a better prognosis than that in the knee joint. Only 3 of 13 hip arthroplasty patients had complications, and all patients who experienced hip arthroplasty presented with a greatly improved Harris score after surgery. All 3 TKA patients had complications, and one of these showed a decreased KSS score. TKA may warrant more specialized care due to the risks of arthroplasty complications [10]. Reoperation for any cause, post-operative infection, or revision arthroplasty may lead to an increased risk of knee arthroplasty failure performed during an oncological process [11].

For the treatment of metaphyseal sarcoma around the knee, Zhang et al. [12] compared child epiphyseal preservation surgery patients with child patients who underwent tumor resection with knee joint arthroplasty. The functional outcomes of the lower limb showed no significant differences between these two groups. There was a risk of tumor recurrence in both groups. However, our patients did not have any obvious tumor recurrence in the local position, which may be due to the benign lesion of TIO disease and unique tumor position. Houdek et al. [11] concluded that malignant tumors would increase the risk of revision, re-operation and post-operative infection following endoprosthetic reconstruction of the knee, while benign tumors would decrease it.

The difference between joint replacement and other surgical treatments including curettage and segmental resection deserves discussion. Wang et al. [4] indicated that although segmental resection could ensure complete resection of the tumor, it would destroy the anatomic structure of the joint. To solve this problem, an artificial joint prosthesis or allogeneic bone segment should be followed to reconstruct the structure. In contrast, curettage can preserve the structure of the joint as much as possible; however, the possibility of recurrence or incomplete resection after curettage is higher than that after segmental resection because tumor cells often infiltrate between bone trabeculae without the presence of a capsule, which makes it difficult to distinguish the tumor boundaries clearly during surgery. As a result, tumor cells can more easily remain. Joint replacement is similar to segmental resection in term of surgical procedure. First, segmental resection is performed to ensure that the tumor is completely removed, and the prosthesis is then implanted to help recover the structure and function of the joint. Consequently, joint replacement has merits of both curettage and segmental resection, namely, preservation of the structure and a low recurrence rate. As the metaphysis around the articular surface plays an important role in the behavior of the joint, based on the merits mentioned above, joint replacement appears to be more suitable for the treatment of tumors located in the metaphysis.

The prognosis of TIO was thought to be excellent following complete resection of the causative neoplasm, and clinical symptoms regularly resolved within a few weeks [2]. For unresectable tumors, curative intended radiotherapy and medical treatment are recommended [13,14].

Arthroplasty in special positions appeared to achieve better therapeutic effects for osteomalacia. Bone metabolism indexes, including alkaline phosphatase, serum phosphorus and serum calcium, were ameliorated after surgery. Alkaline phosphatase indicated a tendency toward bone dissolving resulting from osteocyte splitting release [15]. At approximately 7 days after surgery, serum phosphorus substantially increased with a corresponding decrease in serum calcium. The serum indicators suggest that arthroplasty treatment could quickly recover function after tumor resection.

Symptoms such as bone pain and fatigue showed relief after surgery in most patients. Osteoporosis could return to a normal level in some patients within one year of follow up. From these perspectives, arthroplasty on a tumor around a joint could more positively influence osteomalacia.

## Conclusion

Joint arthroplasty including tumor-expanding resection appears to be a safe and appropriate method for the treatment of TIO patients with a neoplasm located in the metaphysis around the articular surface.

## Supporting information

S1 STROBE Checklist The STROBE checklist of this study. The STROBE checklist was prepared as supporting information.
(DOCX)

S1 Fig The MRI imaging of the 16^th^ patient. On MRI imaging of the knee joint, the tumor was found near the articular surface.
(JPG)

S2 Fig Another view of the 16^th^ patient’s knee joint on MRI. This picture also shows that the tumor was near the articular surface.
(JPG)
