# Supplementary figures and images for: Total hip/knee arthroplasty in the treatment of tumor-induced osteomalacia patients: More than 1 year follow-up
Source: PLoS One. 2017 May 17;12(5):e0177835. doi: 10.1371/journal.pone.0177835 (PMC5435341; doi:10.1371/journal.pone.0177835)

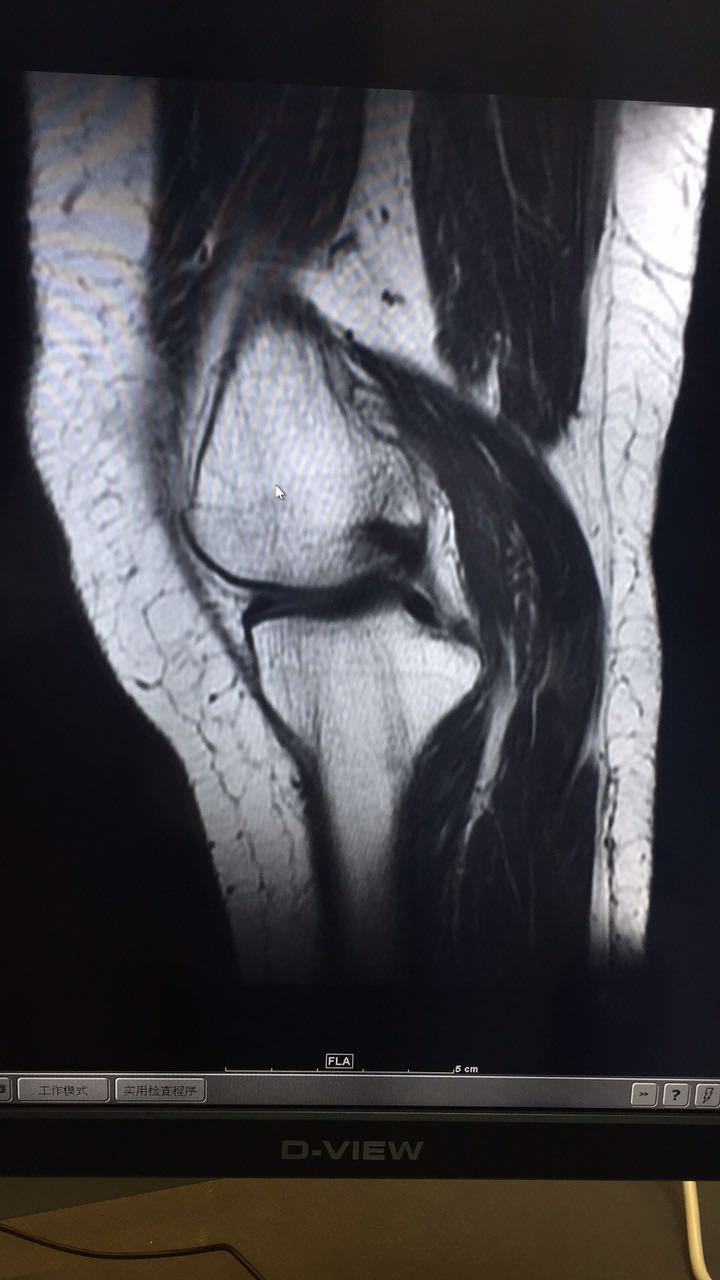

Supplement: S1 Fig — On MRI imaging of the knee joint, the tumor was found near the articular surface. (JPG) [file pone.0177835.s002.jpg]

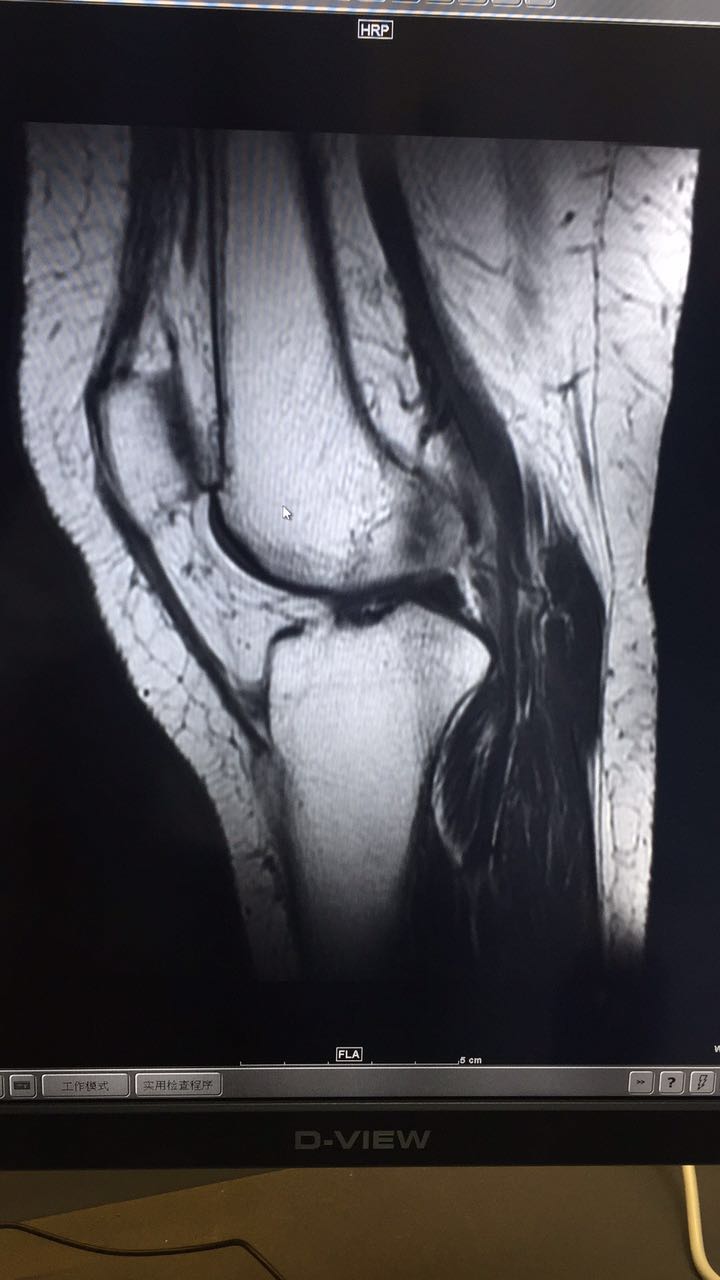

Supplement: S2 Fig — This picture also shows that the tumor was near the articular surface. (JPG) [file pone.0177835.s003.jpg]
